# Supplementary material for: PharmaKU: A Web-Based Tool Aimed at Improving Outreach and Clinical Utility of Pharmacogenomics
Source: J Pers Med. 2021 Mar 16;11(3):210. doi: 10.3390/jpm11030210 (PMC7998233; doi:10.3390/jpm11030210)
Supplement: Supplementary file 1 [file jpm-11-00210-s001.zip › SupplementaryFiles/SampleReport.pdf]

PharmaKu – Personal Pharmaco Genomics Report

Name/Description:

Uploaded VCF: 11369.vcf

Date: 31/12/2020 08:47

Gene	Genotype	Allele Functionality	Phenotype	Clinical Recommendation*
CYP2C19	*1/*17	1 Normal function and 1 Increased function	CYP2C19 Rapid Metabolizer	Adjust Dose
CYP2C9	*1/*1	2 Normal function	CYP2C9 Normal Metabolizer	Usual Dose
CYP2D6	*2/*119	1 Normal function and 1 Unknown function	Indeterminate	
TPMT	*1/*1	2 Normal Function	TPMT Normal Metabolizer	Usual Dose
UGT1A1	*60/*60	2 Normal function		
SLCO1B1	*1/*1B	2 Normal function		
CYP2B6	*1/*1	2 Normal function	CYP2B6 Normal Metabolizer	Usual Dose
CYP3A5	*3/*3	2 No function	CYP3A5 Poor Metabolizer	Adjust Dose
NUDT15	*1/*1	2 Normal Function	NUDT15 Normal Metabolizer	Usual Dose

* See below for the drug dosage adjusments

Interpretation - Consult Note

CYP2C19

This result signifies that the patient has one copy of a normal function allele and one copy of an increased function allele. Based on the genotype result this patient is predicted to be a rapid metabolizer of CYP2C19 substrates. This patient may be at risk for an adverse or poor response to medications that are metabolized by CYP2C19. To avoid an untoward drug response, dose adjustments or alternative therapeutic agents may be necessary for medications metabolized by CYP2C19. Please consult a clinical pharmacist for more information about how CYP2C19 metabolic status influences drug selection and dosing.

Voriconazole

"Pediatrics: Based on the genotype result, this patient is predicted to be a CYP2C19 Rapid Metabolizer and the probability of attainment of therapeutic concentrations is variable. Initiate therapy with recommended standard of care dosing. Meticulous therapeutic drug monitoring to titrate dose to therapeutic trough concentrations is critical due to large variability in trough concentrations in rapid metabolizers.

Adults: Based on the genotype result, this patient is predicted to be a CYP2C19 Rapid Metabolizer and the probability of attainment of therapeutic concentrations is modest. Choose an alternative agent that is not dependent on CYP2C19 metabolism as primary therapy instead of voriconazole such as isavuconazole, liposomal amphotericin B or posaconazole. Selection of an alternative agent should be based upon other clinical factors such as drug interactions, hepatic and/or renal function, suspected or document fungal species, site of infection, and comorbidities. "

Drugs that are affected by CYP2C19:

Amitriptyline, Citalopram, Clopidogrel, Escitalopram, Voriconazole, Sertraline, Clomipramine, Dexlansoprazole, Doxepin, Esomeprazole, Imipramine, Lansoprazole, Omeprazole, Pantoprazole, Rabeprazole, Trimipramine, Brivaracetam, Carisoprodol, Clobazam, Diazepam, Flibanserin

CYP2C9

This result signifies that the patient has two copies of a normal function allele. Based on the genotype result, this patient is predicted to be a CYP2C9 Normal metabolizer. Based only on the CYP2C9 genotype, there is no reason to adjust the dose of most medications that are affected by CYP2C9. Please consult a clinical pharmacist for more specific information about how CYP2C9 function influences drug dosing.

Phenytoin

HLA-B*15:02 genotype may be important for phenytoin adverse events. An HLA-B*15:02 genotype does not appear to have been ordered for this patient. Use of an alternative antiepileptic may be recommended. Please consult a clinical pharmacist for more information.

Drugs that are affected by CYP2C9:

Phenytoin, Warfarin, Siponimod, Tafenoquine, Acenocoumarol, Celecoxib, Flurbiprofen, Diclofenac, Flibanserin,

Lesinurad

CYP2D6

No interpretation available

Drugs that are affected by CYP2D6:

Amitriptyline, Atomoxetine, Codeine, Fluvoxamine, Nortriptyline, Ondansetron, Paroxetine, Tamoxifen, Clomipramine, Desipramine, Doxepin, Imipramine, Trimipramine, Oxycodone, Pitolisant, Tramadol, Tropisetron, Aripiprazole, Brexpiprazole, Dextromethorphan, Eliglustat, Mirtazapine, Pimozide, Protriptyline, Quinidine, Risperidone, Sertraline, Venlafaxine, Vortioxetine, Donepezil, Iloperidone, Methylphenidate, Perphenazine, Carvedilol, Cevimeline, Clozapine, Darifenacin, Dolasetron, Duloxetine, Fesoterodine, Flecainide, Flibanserin, Fluoxetine, Haloperidol, Metoprolol, Modafinil, Olanzapine, Palonosetron, Propafenone, Propranolol, Quinine, Tamsulosin, Terbinafine, Tetrabenazine, Thioridazine, Timolol, Tiotropium, Tolterodine, Zuclopenthixol, Galantamine

TPMT

No interpretation available

Azathioprine

No recommendations available

Mercaptopurine

No recommendations available

Thioguanine

No recommendations available

Drugs that are affected by TPMT:

Azathioprine, Mercaptopurine, Thioguanine

UGT1A1

No interpretation available

Drugs that are affected by UGT1A1:

Irinotecan, Belinostat, Dolutegravir, Pazopanib, Nilotinib, Atazanavir

SLCO1B1

No interpretation available

Drugs that are affected by SLCO1B1:

Cerivastatin, Simvastatin, Methotrexate, Pravastatin, Rosuvastatin

CYP2B6

This result signifies that the patient has two copies of normal function alleles. Based on the genotype result

this patient is predicted to be an normal metabolizer of CYP2B6 substrates. There is no reason to selectively adjust the dose of most medications that are metabolized by CYP2B6. Please consult a clinical pharmacist for more information about how CYP2B6 metabolic status influences drug selection and dosing.

Efavirenz

No recommendations available

Drugs that are affected by CYP2B6:

Efavirenz, Methadone, Nevirapine

CYP3A5

This result signifies that the patient has two copies of a no function allele. Patients with this genotype are expected to require standard tacrolimus dosing. Please consult a clinical pharmacist for more specific dosing information.

Drugs that are affected by CYP3A5:

Tacrolimus, Atazanavir, Cyclosporine, Midazolam, Sirolimus

NUDT15

No interpretation available

Thioguanine

No recommendations available

Drugs that are affected by NUDT15:

Azathioprine, Thioguanine, Mercaptopurine
